# Association between COVID-19 preventive behavioral changes and anxiety in Karachi, Pakistan: A cross-sectional pilot study

**DOI:** 10.1016/j.amsu.2022.103805

**Published:** 2022-05-20

**Authors:** Hyder Ali, Mohammed A. Mamun, Naveed Gianchand, Atia Aijaz, Komal Samir, Sabeen Hyder, Amir H. Pakpour, Irfan Ullah, Muhammad Sohaib Asghar

**Affiliations:** a Dr. Ruth KM Pfau Civil Hospital Karachi, Pakistan; b Dow University of Health Sciences Karachi, Pakistan; c CHINTA Research Bangladesh, Savar, Dhaka, 1342, Bangladesh; d Department of Public Health and Informatics, Jahanirnagar University, Savar, Dhaka, 1342, Bangladesh; e Social Determinants of Health Research Center, Research Institute for Prevention of Non-Communicable Diseases, Qazvin University of Medical Sciences, Qazvin, Iran; f Kabir Medical College, Gandhara University Peshawar, Pakistan; g Naseer Teaching Hospital, Peshawar, Pakistan

**Keywords:** COVID-19, Psychological impacts, Anxiety, Preventive behavior, Hygiene practice, Pakistan

## Abstract

**Background:**

COVID-19 has turned into emergent psychological impacts across cohorts with devastating consequences related to preventive measures. Health organizations recommended some preventive measures (e.g., wearing masks, frequent handwashing, etc.) to overcome the COVID-19 pandemic. However, performing these behaviors may increase anxiety among populations. Thus, the present study aimed to investigate the role of behavioral changes to prevent COVID-19 infection and anxiety during the COVID-19 pandemic in Pakistan.

**Subjects and methods:**

The present cross-sectional study was conducted for 10 days during July 2020 among the general public of Karachi after the imposition of lockdown amid the COVID-19 pandemic, with a sample size of 331 participants. The questionnaire consisted of three parts i.e., (i) socio-demographics, (ii) perception and preventive behaviors towards COVID-19, and (iii) anxiety-related questions using the Urdu Generalized Anxiety Disorder (GAD-7). The data was analyzed using logistic regression to investigate the association between behavior change and anxiety.

**Results:**

Almost half of the participants (i.e., 48.9%) reported being anxious. Although most of the participants were compliant with preventive behavioral changes in their daily lives but no associations between preventive behaviors and anxiety were found. There were significant associations between anxiety and some of the socio-demographic variables (i.e., gender: females were more anxious; age group and marital status single participants were more anxious).

**Conclusion:**

Based on the present findings, it is clearly evident that Pakistani people are suffering psychiatric problems during the COVID-19 pandemic. Hence, appropriate initiatives should be adopted as soon as possible. Besides, COVID-19 related preventive behavioral measures are highly recommended to practice without putting anything back for psychological fears.

## Highlights

• This study highlighted the role of behavioral changes to prevent COVID-19 infection and anxiety.
• Anxiety was found associated with gender, age, and marital status.
• Appropriate measures should be undertaken to control COVID-19 related preventive behavioral practices due to psychological fear.

## 1 Introduction

The recent coronavirus disease (COVID-19) outbreak standing along with its’ pandemic family such as SARS and MERS are another addition to the suffering of fever, cough, fatigue, breathing difficulties, etc. [1]. In Pakistan (where the present study is carried out), the number has surpassed over 954,743 infected patients as of June 28, 2021 and increasing at great pace day by day [2].

China, being the first country under the rage of novel coronavirus, has responded positively with a number of preventive strategies. These strategies include lockdown, quarantine, isolation, hygiene practices, frequent hand washing, etc. Which seem to have positive influences on decreasing the massive and community-based transmission [1,3,4]. Although these strategies have a good impact on predisposing the transmission rates, their sought-after psychological consequences are reported globally. For example, Dsouza et al. [3] reported uprising suicide occurrences because of loneliness, unable to come back home due to lockdown, social boycott and pressure to be quarantine, unavailability of alcohol, etc. Besides, there is evidence that suicide completion has increased as of the decreased quality of life turning to the COVID-19 crisis. Utilizing COVID-19 suicide cases, wide-ranging causative factors related to COVID-19 pandemic are suggested from global studies [3,[5], [6], [7], [8], [9], [10]]. Of these factors, psychological suffering such as fear of COVID-19 infection (i.e., arguably very similar to anxiety, which is studied in the present study) is accounted as the most prominent suicide risk factor [11,12].

Anxiety disorders interplay between threatening signals and the application of safety signals (i.e., the more the victim feels anxious, the more he practices to feel safe). And hence reflect the direct relation between anxiety and behavioral measures [12,13]. Such types of psychiatric disorders are indirect effects of these pandemic situations and are observed quite frequently among natives of different countries [14,15]. Psychiatric disorders along with anxiety are observed globally during these pandemic crises and reported in different regions although, there are limited studies on behavior changes in respect to prevention of the virus roles in psychiatric problems [15,16]. Consistent with other parts of the world, general public residing in Karachi are also coping with the preventive behaviors including hand washing, reduced hangouts with frequent health faculty visits despite this positive behavior there may a wave of anxiety among them, and other psychological impacts as well [17]. In Pakistan, there are a few studies assessing the mental health aftermath of the COVID-19 pandemic [[18], [19], [20]]; and no studies assessing the role of the preventive behavior in psychiatric sufferings like anxiety are ever conducted. However, anxiety in relation to socio-demographics and preventive behavior-related changes is for the first time studied in the present study.

## 2 Methods

### 2.1 Study site, participation and ethics

The present study was conducted in Karachi, the capital of Pakistan within a 10-days of period after the lockdown was imposed (i.e., from 20 to July 30, 2020). The online survey approached the participants with snowball sampling which include initial sets of 4 groups ensuring a broad range of age, gender, occupation, and education. Each volunteer was asked to choose five people they consider suitable for the survey and to send them the questionnaire. Further participants were reached out in the same way until data saturation. The inclusion criteria for participating were - (i) voluntary willingness to participate, (ii) being educated at least primary level, and (iii) being permanent Karachi resident. The study followed highest degree of ethical perspective suggested by Helsinki Declaration, 1975. Besides, the research protocol was registered with the institutional review board of Dow University of Health Sciences, Pakistan (UIN: IRB/DUHS/2020/649). Informed consent was obtained and other ethical issues are highly practiced in the study.

### 2.2 Sample size estimation

Sample size was calculated using ‘www.openepi.com’ website. About 27% moderate to severe anxiety prevalence rate based on a Chinese study, the neighboring country of Pakistan, was considered for the reference value [21]. Moreover, 95% confidence interval and 5% absolute precision were considered for the sample size estimation. Our estimated sample size was 232 considering the total population of 1011 respondents from the parent article.

### 2.3 Measures

The questionnaire consisted of three parts i.e., (i) socio-demographics, (ii) perception and preventive behaviors towards COVID-19, and (iii) anxiety-related questions. The common socio-demographic variables such as age, sex, marital status, education, and occupation were asked. COVID-19 preventive behaviors related to the hygiene practices (e.g., hand washing issues, taking hot baths, etc.) and behavior changes (e.g., mask-wearing, etc.) were also asked in the survey. Lastly, for assessing the anxiety, Urdu version of the Generalized Anxiety Disorder (GAD-7) scale was used [22]. The scale has 7-items with a 4-Likert points (i.e., *not at all sure* to *nearly every day*) donating a possible 0 to 21 scores, whereas 10 is the cutoff score of possible for anxiety [23,24]. Besides, the severity of anxiety is denoted as mild [[5], [6], [7], [8], [9]], moderate [[10], [11], [12], [13], [14]], and severe [[15], [16], [17], [18], [19], [20], [21]].

### 2.4 Statistical analysis

All the collected information was assessed and analyzed via the software Statistical Package for Social Sciences (SPSS) version 24. Chi-square tests were used to see the anxiety associations with socio-demographic and hygiene practices and behavior changes related variables. Fisher exact tests were used in case each cell had less than 20% expected value. Besides, binary logistic regression was performed to see the odds ratio with 95% confidence interval of the variables.

## 3 Results

Of the total 331 respondents, 52.0% of them were males and the age range was 13–79 years (mean age = 30.44 ± 11.604 years). About 58.0% of the participants were single in relationship, while most of them were either at undergraduate (47.1%) or post-graduate level (50.8%). Within occupation, 43.8% were students, whereas the rest were health care providers (39.6%) and others (Table 1).

**Table 1:** Association between socio-demographics and anxiety.

Parameters	Anxious (48.94%, n = 162)	Normal (51.06%, n = 169)	*p*-value	Odds Ratio (95% C. I.)
Gender				
Male (172; 52.0%)	68 (39.5)	104 (60.5)	<0.001	0.452 (0.291–0.702)
Female (159; 48.0%)	94 (59.1)	65 (40.9)		Reference
***Age***				
<20 (24; 7.3%)	15 (62.5)	9 (37.5)	0.049	Reference
21-30 (190; 57.4%)	96 (50.5)	94 (49.5)		0.613 (0.256–1.468)
31-40 (55; 16.6%)	30(54.5)	25 (45.5)		0.720 (0.270–1.923)
41-50 (36; 10.9%)	10 (27.8)	26 (72.2)		0.231 (0.077–0.695)
>51 (26; 7.9%)	11 (42.3)	15 (57.7)		0.440 (0.141–1.369)
***Marital status***				
Single (192; 58.0%)	106 (55.2)	86 (44.8)	0.007	Reference
Married (139; 42.0%)	56 (40.3)	83 (59.7)		1.827 (1.174–2.844)
***Education status***				
Secondary & Higher Secondary (7; 2.1%)	4 (57.1)	3 (42.9)	0.833	Reference
Undergraduate (156; 47.1%)	78 (50.0)	78 (50.0)		1.467 (0.318–6.755)
Postgraduate (168; 50.8%)	80 (47.6)	88 (52.4)		1.10 (0.711–1.701)
***Occupation***				
Student (145; 43.8%)	75 (51.7)	70 (48.3)	0.617	Reference
Healthcare professional (131; 39.6%)	60 (45.8)	71 (54.2)		0.789 (0.491–1.266)
Others (55; 16.6%)	27 (49.1)	28 (50.9)		0.900 (0.484–1.675)

Note: In marital status, the divorced frequency was less than 5 so that were added in unmarried category.

**Fig. 1:**
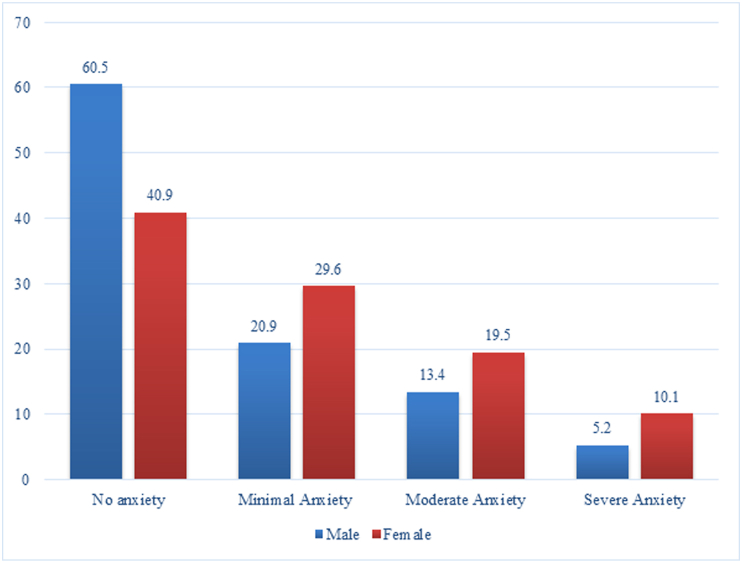
Distribution of anxiety severity across gender (χ2=13.113; p <0.001)

The mean score of anxiety was 5.70 ± 5.272 across all the participants, and 48.9% of them were reported to have anxiety. Within gender, female participants were more likely to be anxious compared to male encounters (*p* < 0.001). Fig. 1 represents the anxiety severity distribution across gender (χ^2^ = 13.113; *p* < 0.001). Besides, age group is significantly associated with anxiety, i.e., the highest anxiety rate was reported in 41–50 years of age groups, followed by > 51 years, 21–30 years, etc. (*p* < 0.05). People being single are less likely to be anxious that the married ones (44.8% vs. 59.7%; *p* = 0.007). Lastly, education level and occupation were not associated with anxiety (Table 1).

Preventive behaviors toward COVID-19 are reported in Table 2. Overall frequent hand washing or mask use was reported by approximately all of the participants (i.e., 98.2%). Besides, 57.1% of the participants took a hot bath daily, whereas 37.5% rose their nose daily, 74.0% took excess vitamin C products, and 81.3% wore mask while going outside. None of the preventive behaviors were associated with anxiety status (Table 2).

**Table 2:** Association between COVID-19 related behavioral changes and anxiety.

Parameters	Anxious (48.94%, n = 162)	Normal (51.06%, n = 169)	*p*-value	Odds Ratio (95% C. I.)
Either washing hands or using face masks frequently				
Yes (325; 98.2%)	158 (48.6)	167 (51.4)	0.440	0.473 (0.085–2.619)
No (06; 1.8%)	4 (66.7)	2 (33.3)		Reference
***Take hot bath daily***				
Yes (189; 57.1%)	98 (51.9)	91 (48.1)	0.222	1.313 (0.848–2.031)
No (142; 42.9%)	64 (45.1)	78 (54.9)		Reference
***Rinse the nose with saline***				
Yes (124; 37.5%)	66 (53.2)	58 (46.8)	0.228	1.316 (0.842–2.056)
No (207; 62.5%)	96 (46.4)	111 (53.6)		Reference
***How frequent wash hands per day***				
3-5 times (33; 10%)	13 (39.4)	20 (60.6)	0.158	Reference
6-8 times (114; 34.4%)	59 (51.8)	55 (48.2)		1.650 (0.750–3.633)
9-11 times (97; 29.3%)	41 (42.3)	56 (57.7)		1.126 (0.503–2.522)
12-14 times (87; 26.3%)	49 (56.3)	38 (43.7)		1.984 (0.877–4.490)
***How long it takes to wash hands***				
<10 seconds (51, 15.4%)	28 (54.9)	23 (45.1)	0.117	Reference
11-20 Seconds (239, 72.2%)	121 (50.6)	118 (49.4)		0.842 (0.459–1.546)
21–40 seconds (35, 10.6%)	11 (31.4)	24 (68.6)		0.376 (0.153–0.928)
41-60 Seconds (06, 1.8%)	2 (33.3)	4 (66.7)		0.411 (0.69–2.447)
***Wearing mask when going outside***				
Yes (269, 81.3%)	133 (49.4)	136 (50.6)	0.705	1.113 (0.640–1.935)
No (62, 18.7%)	29 (46.8)	33 (43.2)		Reference

The participants were enquired about their perception regarding usage of a facemask by healthy ones is a waste of source or not and 258 (77.9%) have disagreed with the statement while only 73 (22.1%) appreciated the use of masks by patients only. Most of the participants (95.5%) were aware of the symptoms of presenting a global emergency and the responses with respect to symptoms were collected. Luckily, a large number of participants (i.e., 76.4%) had not noticed any of the symptoms of infection in anyone. A small number of participants 51(15.4%) noted such symptoms in themselves while 18 (5.4%) noted in their family members. None of the symptoms-related questions were associated with anxiety (Table 3).

**Table 3:** Association between COVID-19 related symptoms and anxiety.

Parameters	Anxious (48.94%, n = 162)	Normal (51.06%, n = 169)	*p*-value	Odds Ratio (95% C. I.)
Are you aware of symptoms of COVID-19?				
Yes (316, 95.5%)	152 (48.1)	164 (51.9)	0.160	2.158 (0.721–6.457)
No (15, 4.5%)	10 (66.7)	5 (33.3)		Reference
***Do you think that consumption of face-mask along with other safety measures by healthy person is wastage of source?***				
Disagree (258, 77.9%)	124 (48.1)	134 (51.9)	0.547	1 (0.547–1.173)
Agree (73, 22.1%)	38 (52.1)	35 (47.9)		Reference
***Kindly, let us know within last 2 weeks have you ever noticed any of the above symptoms among given options?***				
Yourself (51, 15.4%)	33 (64.7)	18 (35.3)	0.068	0.545 (0.032–9.249)
Haven't noticed any symptoms (253, 76.4%)	114 (45.1)	139 (54.9)		1.219 (0.075–19.710)
Friends (07, 2.1%)	3 (42.9)	4 (57.1)		1.333 (0.057–31.121)
Family member (18, 5.4%)	11 (61.1)	7 (38.9)		0.636 (0.034–11.909)
Other (02, 0.6%)	1 (50.0)	1 (50.0)		Reference

## 4 Discussion

COVID-19 has turned emergent psychological impacts across cohorts with devastating consequences [25,26]. Thus, the World Health Organization is well aware of the collateral mental health damages caused by any typical pandemic and issued a general consideration regarding mental wellbeing during the present COVID-19 outbreak [27]. Aggravated threats to mental wellbeing are not uncommon specifically in developing countries like Pakistan, whereas rapid mental health assessments are needed to adopt any preventive strategies [9,[28], [29], [30]].

A recent Pakistani study showed that 34% of the students from higher educational institute suffer from anxiety during the COVID-19 period [31], and another previous review article back from non-COVID period also reported a similar anxiety prevalence rate among the general Pakistani people [32]; which is quite low with respect to this study prevalence rate (i.e. 48.9%). Based on Qian et al. [33] findings, 32.7% and 20.4% anxiety rates are reported from Wuhan and Shanghai, China respectively. Whereas the rate is 21.6% in a UK sample [34], around 34.2% among Bangladesh adult sample [35] and 12.05% reported from the Indian context [36]. Recently, Vindegaard and Benros [15] reviewed the prevalence of mental disorders across various groups. The highest prevalence of mental problems among COVID-19 patients (96.2% post-traumatic stress symptoms, 29.2% depression), preceded by patients with psychiatric disorders prior to and during COVID-19 (37.5% of patients with pre-existing psychiatric disorders reported worsening symptomatology of their eating disorder, 56.2% symptoms of anxiety, 20.9% of patients with pre-existing psychiatric disorders with worsening symptoms). In addition, 23.2% of healthcare professionals reported anxiety overall, 22.8% depression, and 38.9% insomnia [37]. However, the present study found the prevalence rate to 48.9%, which is much high than any other studies aftermath the pandemic.

To the nature of gender, female is more vulnerable to mental suffering and in the sense of pandemic, females are being reported to have higher psychiatric suffering and this may be because of their lower coping capacity as well as resilience [19,21,38]. After imposing lockdown, male partners are supposed to stay at home, which leading more domestic violence. Inter-partner violence and domestic violence is also being highly reported in Pakistan. For instance, the rate is reported to be 90% of the Pakistani women at the hands of their husbands or families, whereas sexual abuse, particularly domestic rape is reported among 47% of the married women [39]. Half of these women who experienced domestic violence keep silent and only 0.4% of them seek legal action [39]. The report also suggests that (female) participants being married are at the risk of such violence. Similar findings are suggested from the COVID-19 related studies [19]. Thus, the present finding (i.e., female suffers more anxiety) is consistent with the ongoing scenario throughout the world; although single participants (not restricted to women participants only) showed a higher anxiety rate, which may be the effect of including other participants to the analysis. Thus, further studies considering the actual effect of domestic violence is suggested.

Anxiety in relation to age is being highly reported in young adults [40], although the present study supports heterogenous continuation of anxiety rate with the age groups. Besides, education level and profession are not associated with making people more anxious. Although, healthcare professionals are supposed to suffer from more psychiatric problems because of a number of reasons related to COVID-19 patients’ exposure as well as other issues [1,6]. Similarly, with respect to profession and mental health, the Pakistani health care providers are suggested to face its worst impact in the form of anxiety, stress level, fear of infection, lack of safety gear equipment, and distance from the family members and friends with every increasing hour there is an addition to traumatizing impact on mental health among the whole population [41]. However, our finding is not similar to the prior one, this may be because of lower sample size and not considering other wide-ranging occupations; thus, further studies covering this limitation are warranted.

There are huge literatures available on public knowledge and awareness towards COVID-19 pandemic [42,43]. But with respect to good practice with psychological problems, this study provides initial observation for the first time to the authors' best knowledge. As there are no actual COVID-19 treatments, the World Health Organization provided preventive measures (e.g., proper hand hygiene practices by repeated washing with a duration of 20–30 seconds, or taking alcohol rub for keeping themselves virus-free, etc [44]. These measures can be burdensome to some individuals. Besides, other psychological burdens can be turned because of frequent behavioral changes along with restlessness related to social distance and lockdown to escape from the virus infection [45]. But the present findings do not support any of these claims; hence re-investigation considering the present study's limitations is warranted.

The study can be limited because of its cross-sectional nature with a limited sample size. Besides, an online survey may turn the representativeness of the study. Despite these limitations, the study provides an initial observation on preventive behaviors risk to mental health outcome.

## 5 Conclusions

Half of the participants were anxious; there is no significant relationship between anxiety and preventive behaviors although some of the socio-demographics are significantly correlated with it. Based on the present findings, encouraging people to preventive behaviors against the COVID-19 can be suggested as it doesn't increase psychological suffering.

## Provenance and peer review

Externally peer reviewed, not commissioned.

## Ethical approval

Ethical approval was taken in this study from institutional review board of Dow University of Health Sciences (Ref no: IRB/DUHS/2020/649).

## Sources of funding

None.

## Author contribution

M.A.M, H.A, and N.G conceived the idea; A.A, K.S, A.H.P, and S.H collected the data; M.S.A and I.U analyzed and interpreted the data; M.A.M, H.A, N.G, A.H.P, I.U, and A.A did write up of the manuscript; and finally, I.U, K.S, M.S.A and S.H reviewed and revised the manuscript for intellectual content critically. All authors approved the final version of the manuscript.

## Conflicts of interest

None.

## Consent

All participants were given a thorough explanation of the study's purpose and benefits followed by written informed consent.

## Registration of research studies

1. Name of the registry: Dow University of Health Sciences.
2. Unique Identifying number or registration ID: IRB/DUHS//2020/649.
3. Hyperlink to your specific registration (must be publicly accessible and will be checked):

## Guarantor

Muhammad Sohaib Asghar.
